# A minireview on the bioremediative potential of microbial enzymes as solution to emerging microplastic pollution

**DOI:** 10.3389/fmicb.2022.1066133

**Published:** 2023-03-02

**Authors:** Rener De Jesus, Ruwaya Alkendi

**Affiliations:** ^1^College of Graduate Studies, United Arab Emirates University, Al Ain, United Arab Emirates; ^2^Department of Biology, College of Science, United Arab Emirates University, Al Ain, United Arab Emirates

**Keywords:** bioprospecting, bioremediation, plastic biodegradation, biotechnological tools, enzyme activity, microbial chassis

## Abstract

Accumulating plastics in the biosphere implicates adverse effects, raising serious concern among scientists worldwide. Plastic waste in nature disintegrates into microplastics. Because of their minute appearance, at a scale of <5 mm, microplastics easily penetrate different pristine water bodies and terrestrial niches, posing detrimental effects on flora and fauna. The potential bioremediative application of microbial enzymes is a sustainable solution for the degradation of microplastics. Studies have reported a plethora of bacterial and fungal species that can degrade synthetic plastics by excreting plastic-degrading enzymes. Identified microbial enzymes, such as *Is*PETase and *Is*MHETase from *Ideonella sakaiensis* 201-F6 and *Thermobifida fusca* cutinase (Tfc), are able to depolymerize plastic polymer chains producing ecologically harmless molecules like carbon dioxide and water. However, thermal stability and pH sensitivity are among the biochemical limitations of the plastic-degrading enzymes that affect their overall catalytic activities. The application of biotechnological approaches improves enzyme action and production. Protein-based engineering yields enzyme variants with higher enzymatic activity and temperature-stable properties, while site-directed mutagenesis using the *Escherichia coli* model system expresses mutant thermostable enzymes. Furthermore, microalgal chassis is a promising model system for “green” microplastic biodegradation. Hence, the bioremediative properties of microbial enzymes are genuinely encouraging for the biodegradation of synthetic microplastic polymers.

## 1. Introduction

Plastics have a lot of good qualities, including weightless and stable physical properties, making them indispensable and highly resilient materials. The massive plastic production, which exponentially started in the 1950s, and the widespread usage of plastics have resulted in a large volume of post-consumer waste being dumped in landfills or marine environments (Jambeck et al., 2015; Geyer et al., 2017). In a recent United Nations Environment Programme (UNEP) report, around 400 million metric tons of plastic waste were produced annually (UNEP (United Nations Environment Programme), 2021). Experts believe that by 2060, global plastic waste production will be tripled, which half will end up in landfills, and the rest will be distributed in the environment (Organization for Economic Co-operation and Development, 2021). Thus, concrete environmental regulation and waste disposal management should be rationalized to control this impending environmental issue.

Plastic biodegradation is a natural process. Without human interference, the natural breakdown of plastic litter can occur *via* weathering, biodeterioration, and biofragmentation (Ojeda et al., 2011; Miles et al., 2017; Syranidou et al., 2019). However, this innate route is generally gradual. A plastic item can be degraded entirely after a hundred to thousand years (Delaney, 2013). Weathered or fragmented plastic items are significant sources of plastic particles called microplastics (MPs; Ter Halle et al., 2016; Kalogerakis et al., 2017). MPs are polymers with a size of <5 mm (about 0.2 in). MPs can be categorized into primary or secondary MPs (Arthur et al., 2009; Lehtiniemi et al., 2018). The primary MPs are product additives found in personal care and cosmetics (Habib et al., 2020), paint coatings, cleaning agents, and tire wear (Verschoor et al., 2016), to name a few. While secondary MPs originate from degraded plastic waste, such as water bottles (Winkler et al., 2019) and carry bags (Yurtsever and Yurtsever, 2018). Despite their different origins, both primary and secondary MPs are suspended in open waters (Han et al., 2020), in water columns (Choy et al., 2019), or embedded in the soil (Liu et al., 2018). MPs are chemically derived from various synthetic polymers, *viz.* polyethylene (PE), polypropylene (PP), polyvinyl chloride, polystyrene (PS), and polyamide (PA; Lise Nerland et al., 2014; Lin et al., 2018), that appear in different morphologies (i.e., fibers, fragments, beads), colors, and length. Because of their minute size, MPs cause detrimental effects on flora and fauna (Cao et al., 2017; Rodriguez-Seijo et al., 2017; de Souza Machado et al., 2019).

Bioprospecting is a process that explores biological products from plants, animals, and microorganisms (Ramesha et al., 2011). Bioprospecting offers a sustainable solution to many impending environmental issues like microplastic pollution. Many microorganisms can secrete enzymes with bioremediative potential against plastic particles (Table 1). These enzymes have shown remarkable biodegradation against various polymers and toxic compounds (Bhandari et al., 2021). The current waste disposal practices are inadequate in regulating litter quantities. As a result, there is a snowballing interest in exploiting efficient microbes to degrade many types of plastic. Therefore, this minireview paper focuses on the microbial enzymes involved in plastic polymer biodegradation, which offers a ‘bird’s-eye view’ of the bioremediative potential to assimilate microplastics.

**Table 1:** List of some reported plastic-degrading enzymes from various microbial strains against various polymer types.

Microbial strain	Source/Sample type	Identified	Molecular weight (kDa)	Polymer type	Size	Ref
		enzyme			(mm)	
*Amycolatopsis orientalis* ssp. *orientalis*	Culture collection	PLAase I	24	PLA powder and microfilm	0.3–0.5	Li et al. (2008)
		PLAase II	19.5			
		PLAase III	18			
*Aspergillus flavus* PEDX3	Wax moth gut	Laccase-like multicopper oxidases	–	LDPE	<0.2	Zhang et al. (2020)
*Bacillus subtilis*	Soil	Polyurethanase	28	Impranil DLN (PU)	0.002	Rowe and Howard (2002)
*Humicola insolens*	Commercial product (Novozym© 51,032)	Cutinase	32	PET particles	5	Carniel et al. (2017)
*Ideonella sakaiensis* 201-F6	PET bottle recycling site	PETase	24	PET film	6	Yoshida et al. (2016)
*Pseudomonas aestusnigri* VGXO14	Crude oil-polluted marine sand	Hydrolase	32	Impranil DLN-SD (PE-PU)	0.1	Bollinger et al. (2020)
*Synechococcus* sp. PCC 7002	Culture collection	Esterase	-	PE nanosphere	0.0002–0.0099	Machado et al. (2020)
		Hydrolase	-			
*Thermobifida fusca* KW3 (DSM 6013)	Culture collection	Hydrolase TfCut2	-	PET nanoparticles	0.1–0.16	Barth et al. (2015)
		Carboxylesterase TfCa	52.94	PET particles	04-Aug	Billig et al. (2010)
*Thielavia terrestris* CAU709	Soil	Cutinase TtcutA	25.3	PET film	5	Yang et al. (2013)

## 2. Factors affecting plastic biodegradation process

Abiotic-biotic factors have essential roles in the biodegradability of plastics. Abiotic factors, such as temperature, pH, light, and humidity, crucially influence biodegradation (Gewert et al., 2018; Oluwasina et al., 2019; Singh et al., 2019; Arisa-Tarazona et al., 2020). These factors enhance the hydrolysis of plastic polymers leading to chain scission. The scission allows biotic factors (i.e., microorganisms) to further polymer degradation. Temperature affects microbial diversity and activity (Zoungranan et al., 2020). Temperatures over 30°C decelerate plastic breakdown but increase microbial species abundance, which improves the biodegradation rate by 20% (Zoungranan et al., 2020). At the same time, pH promotes microbial growth and enzymatic activity that affects biodegradation. At 0°C with pH 3 and 11, MPs showed brittleness and fragmentations (Arisa-Tarazona et al., 2020). Furthermore, photolysis using UV light improves plastic degradation and applies as a pre-treatment method. Synthetic plastics exposed to UV for 12 months have produced fragments with decreasing sizes (Song et al., 2017). Humidity is a significant environmental factor influencing plastic biodegradability, as well. Humidity may negatively or positively stimulate microbial growth and activity. High moisture content would increase biodegradation, but excessive moisture content hinders biodegradation due to dilution effects (Oluwasina et al., 2019).

Moreover, the overall plastic biodegradation is also affected by the plastic’s surface area and polymer characteristics. High-molecular weight synthetic plastics (e.g., PE and PP) have reduced hydrophilicity because of their intact polymer chains and are thus more difficult to degrade than low-molecular weight plastics (Kawai, 1995). In addition, the absence of functional groups attribute to the durability of plastics. Some plastic additives have pro-oxidant functional groups with hydrophilic characteristics (Chiellini et al., 2006; Harshvardhan and Jha, 2013) and are receptive to attack by microbial enzymes, light, and water. Taken together, the abiotic-biotic factors determine the efficiency of microplastic biodegradation. However, the structural complexity of synthetic polymers affects the actions of these factors. Factors affecting the plastic biodegradation were discussed in many comprehensive review papers (Shah et al., 2008; Tokiwa et al., 2009; Yuan et al., 2020; Shilpa Basak and Meena, 2022).

## 3. Biodegradation of microplastics by microbial enzymes

### 3.1. Microbial enzymes involved in biodegradation of synthetic polymers

Because microorganisms can produce enzymes that enable them to use plastic as a source of energy, microbes are ideal candidates for reducing plastic waste in the environment. Gambarini et al. (2021) identified a substantial number of putative microbial plastic degraders belonging to 12 different microbial phyla, of which just seven phyla have reported degraders to date. It indicates that bacterial and fungal phyla have a significant untapped potential for discovering enzymes that can degrade plastics. In fact, a broad family of microbial enzymes was already been isolated, such as hydrolases, laccases, peroxidases, and lipases that showed degradation of synthetic plastics. Though the differences between fungal and bacterial enzymes are not exclusively discussed in many literatures, their distinct physiologies are likely to differentially influence the rates of plastic biodegradation (Waring et al., 2013). Related studies found that most fungal enzymes have complete enzymatic systems for the depolymerization and mineralization of plastic (Blanchette, 1995; Nikhil et al., 2012; Zhu et al., 2016).

**Figure 1:**
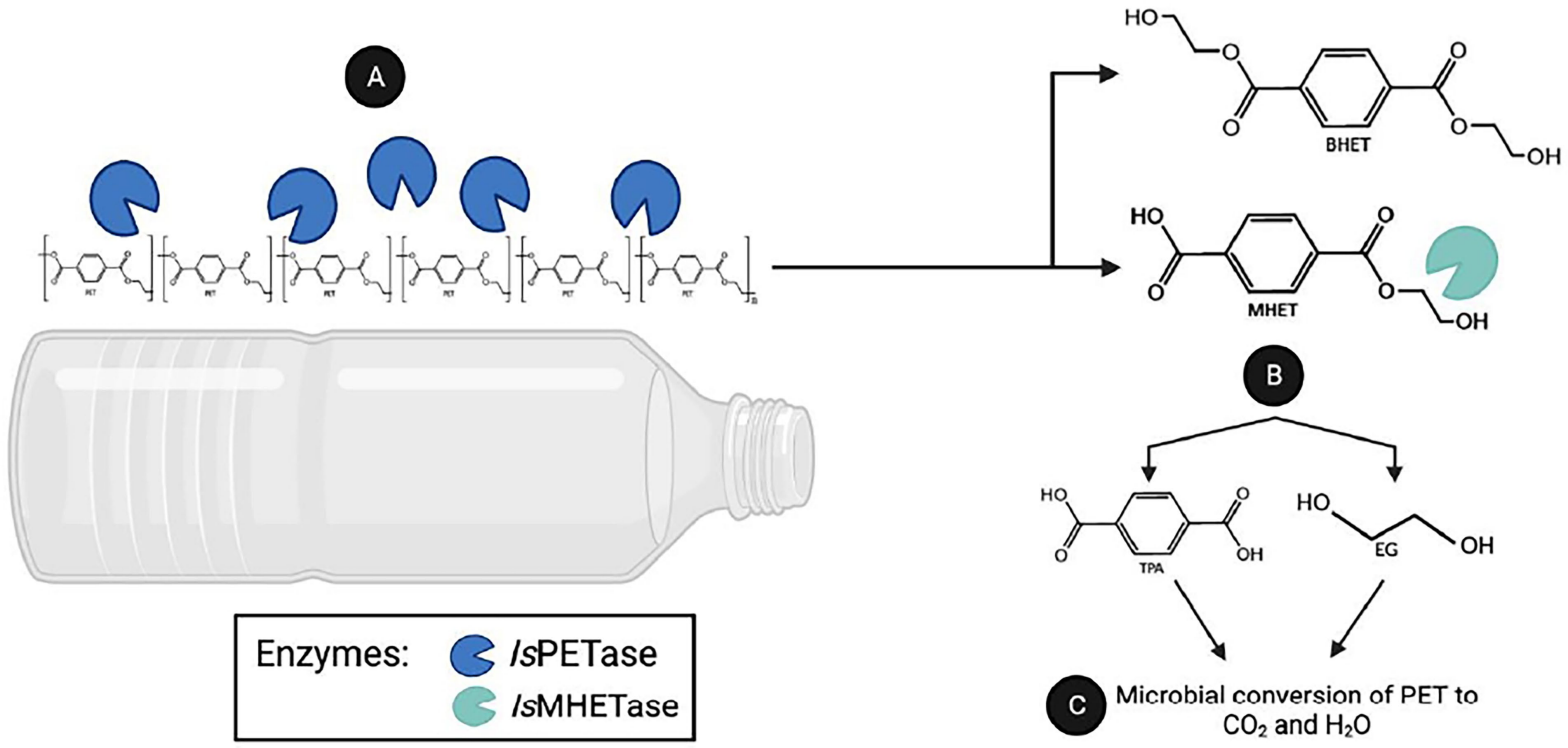
PET degradation by the enzymes PETase and MHETase. The Gram-negative bacterium *I. sakaiensis* 201-F6 is able to produce *Is*PETase and *Is*MHETase enzymes. **(A)** The *Is*PETase degrads polythylene chain producing bis(2-hydroxyethyl)terephthalate(BHET) or mono(2-hydroxyethyl) terepththalate(MHET). **(B)** While *Is*MHETase are converting MHET to non-toxic compounds: terephthalic acid and ethylene glycol. **(C)** By assimilation, these molecules converted to a carbon dioxide as byproduct of microbial conversion of PET.

Since microbial enzymes are generally more stable than their plant and animal counterparts, microbes are gaining interest as a source of beneficial plastic-degrading enzymes. A notable example is the bacterial strain *Ideonella sakaiensis* 201-F6 that can degrade polyethylene terephthalate (PET). PET is one of the most widely used synthetic plastic, with an annual global output of over 50 million tons (Bornscheuer, 2016). The strain 201-F6 produced cutinase-like serine hydrolases named *Is*PETase and *Is*MHETase (Yoshida et al., 2016). The PET degradation process can be divided into two steps: the nick generation step and the terminal digestion step (Joo et al., 2018). In the nick generation step, the *Is*PETase cleaves one ester bond causing the formation of a nick in PET polymer chain, resulting in the generation of two PET chains with different terminals: terephthalic acid (TPA)-terminal and hydroxyethyl (HE)-terminal. Then, in the terminal digestion step, two PET chains having those termini are digested into bis-(2-hydroxyethyl)terephthalic acid (BHET) and mono-(2-hydroxyethyl)terephthalic acid (MHET) monomers (Joo et al., 2018). Subsequent digestion of these molecules, which *Is*MHETase breaks down MHET, produces ecologically harmless terephthalic acid (TPA) and ethylene glycol (EG) by-products (Carniel et al., 2017; Joo et al., 2018; Knott et al., 2020; Figure 1). Through assimilation and mineralization, TPA and EG are converted into carbon dioxide and water. However, one of the limitations of using *Is*PETase is its low thermal stability (Yoshida et al., 2016; da Costa et al., 2021). Nevertheless, because of impressive enzymatic activity against PET, *Is*PETase has been subjected to structural improvements using various biotechnological tools.

A variety of cutinases have been identified to degrade PET as well. Cutinases have been found in fungi and bacteria, such as *Fusarium solani pisi* (Stavila et al., 2013) and *Thermobifida fusca* (Chen et al., 2010). The cutinases from both groups belong to the α/ß-hydrolase superfamily with similar spatial structures, catalytic characteristics, and substrate specificities. Despite the similarities, fungal and bacterial cutinases lack sequence homology. Thus, cutinases can be classified into prokaryotic and eukaryotic cutinase subfamilies (Chen et al., 2008). The *T. fusca* cutinase (Tfc) was reported to improve PET degradation with microbial pre-treatment. Microbial pre-treatment with *Stenotrophomonas pavanii* JWG-G1 reduced PET surface hydrophobicity, causing an easy binding for Tfc (Huang et al., 2022). Microbial pre-treatment could be a novel approach for microplastic biodegradation and to increase the degradation rate. The synergistic action of JWG-G1 and Tfc initially starts with surface binding and biofilm development of JWG-G1, which leads to the formation of functional groups from breaking ester bonds to yield PET oligomers. Like *Is*PETase and *Is*MHETase, Tfc hydrolyzes PET oligomers (Huang et al., 2022) to produce carbon dioxide and water molecules. Furthermore, several microbial strains were reported of producing cutinases with beneficial bioremediative applications. Examples are *Aspergillus* sp. RL_2_Ct (Kumari et al., 2016), *Pseudomonas cepacia* NRRL B-2320 (Dutta et al., 2013), and *Aspergillus nidulans* that produced thermo-alkaline cutinase called ANCUT2 (Bermúdez-Garcia et al., 2017). Cutinases also degrade other polymers, like poly(butylene succinate) (PBS; Hu et al., 2016) and polyester (Baker et al., 2012).

Another family of plastic-degrading enzymes are laccases that primarily described in fungal lignin biodegradation. However, laccases have been identified in both bacterial and fungal species. Laccases are copper-dependent enzymes that perform oxidation reactions of an oxygen molecule to water (Mayer and Staples, 2002; Martínez et al., 2005; Munk et al., 2015). These enzymes showed degradation of PA, PE, and PP (Fujisawa et al., 2001; Sheik et al., 2015). The degradation steps are perhaps similar to the lignin decomposition, which proceeds by oxidative reactions that breaks carbon-to-carbon bonds or ether linkages to liberate functional groups (Asina et al., 2016). Fungal species like *Cochliobolus* sp. (Sumathi et al., 2016), *Phlebia* spp. (Arora and Rampal, 2002), *Podospora anserina* (Xie et al., 2014), and *Yarrowia lipolytica* (Lee et al., 2012) were reported of laccase production and involved in break down of lignin. Bacterial laccases, on the other hand, are more stable at varying conditions, like pH and temperature, than fungal laccases (Chauhan et al., 2017), which indicates an encouraging application in microplastic bioremediation. Soil bacterium *Azospirillum lipoferum* produced laccase-like polyphenol oxidase that was thermostable up to 70°C with optimal pH of 6.0 (Diamantidis et al., 2000). Other strains, such as *Bacillus subtilis* MTCC 2414 (Muthukumarasamy et al., 2015), *Microbulbifer hydrolyticus* IRE-3 (Li et al., 2020), *Pseudomonas extremorientalis* BU118 (Neifar et al., 2016), and *Serratia marcescens* MTCC 4822 (Kaira et al., 2015), were reported of producing laccases with broad deterioration activities against pollutants, including plastics. Nevertheless, the industrial application of laccases is restricted due to some limitations, like low yield and high-cost production (Akpinar and Ozturk Urek, 2017; Chenthamarakshan et al., 2017).

Peroxidases are a large family of oxidoreductases known to catalyze the oxidation of many inorganic and organic substrates by using hydrogen peroxide (Adewale and Adekunle, 2018; Twala et al., 2020). Most of the peroxidases were reported from various fungal species and involved in lignin degradation with laccases. The addition of manganese peroxidase showed increased PE degradation by lignin-degrading fungi (Iiyoshi et al., 1998), which is like the copper-induced laccase activity of IRE-3 (Li et al., 2020), improves biodegradation rates. Trace elements, such as manganese and copper, protect cells from oxidative stress resulting (Bonnarme and Jeffries, 1990; Levin et al., 2002) in the retention of polymer-degrading activities. Marine fungus *Alternaria alternata* FB1 efficiently degraded PE polymers by producing 153 exoenzymes, including peroxidase and laccase, and caused a 95% reduction in the polymer’s molecular weight (Gao et al., 2022). Compared to fungal peroxidase, studies about bacterial peroxidases are limited. Future biodegradation studies of plastics using bacterial peroxidase could open new avenues for breaking down many synthetic plastics.

Together with cutinase and hydrolase, lipase is one of the common enzymes associated with plastic degradation. Lipases have been produced in many bacterial and fungal strains. As discussed earlier, increasing molecular weight hinders the biodegradation rate, and specific fungal lipases can break down high-molecular weight polymers. Lipase is one of the best biocatalysts for PET degradation. Lipase B (CALB) from the yeast *Candida antarctica*, formerly *Pseudozyma antarctica*, is known for its high selectivity and catalytic activity. The action of CALB is similar to *Is*PETase and *Is*MHETase activities (Boneta et al., 2021). CALB demonstrated high-efficiency hydrolysis steps and polymer scission that led to the accumulation of TPA (Carniel et al., 2017; de Castro and Carniel, 2017; de Castro et al., 2017). Moreover, CALB and *Humicola insolens* cutinase resulted in complete PET depolymerization with a mole fraction of up to 0.88 and a 7.7-fold increase in PET to yield TPA (Carniel et al., 2017). Another, a purified lipase (CLE) from the *Cryptococcus* sp. strain S-2, effectively hydrolyzed high-molecular weight plastic polymers like poly (lactic acid) and other bio-based polymers, such as polybutylene succinate, poly (ɛ-caprolactone), and poly (3-hydroxybutyrate), at a concentration of 0.8 μg/ml in 88 h (Masaki et al., 2005). It was found that the hydrolytic action involves the activation of a catalytic triad following the formation of tetrahedral intermediates that stabilizes the enzyme structure (Yoshitake et al., 2009). Hydrolysis produced volatile fatty acids and glycerols that eventually assimilated by the microorganisms to yield lipid acyl chains for cell membrane maintenance (Poddar et al., 2020). Bacterial lipases are also recognized for breaking down plastic polymers like polyurethane (Gautam et al., 2007) and PET oligomers (Swiderek et al., 2022). Various bacterial species were reported to secrete novel lipases, including those from thermophilic and psychrophilic strains (Mobarak-Qamsari et al., 2011; Rabbani et al., 2013; Maiangwa et al., 2014). Members of mesophilic *Bacillus* spp. and *Pseudomonas* spp. have been described to produce lipases (Elwan et al., 1983; Khannous et al., 2014; Jaiswal et al., 2017) with potential microplastic biodegradation. However, the utilization of these enzymes in microplastic biodegradation has not been extensively explored. Further studies should be performed on the possible polymer degradation and assimilation of degradation by-products.

### 3.2. Advanced biotechnological approaches to enhance enzyme actions against microplastics

In order to overcome the possible limitations of microbial enzymes in the biodegradation of MPs, new strategies need to be created. Researchers have recently demonstrated that biotechnological strategies improve enzyme structure and stability. One of the biotechnological tools widely used in protein engineering—the structural-based modeling, yields enzyme variants with higher enzymatic activity and temperature-stable properties. Son et al. (2020) successfully created *Is*PETase^S121E/D186H/S242T/N246D^ variant with enhanced substrate binding affinity and thermo-stable characteristics (Ma et al., 2018; Son et al., 2020; Meng et al., 2021). These enzyme variants exhibited 58-fold greater activity than the wild-type *Is*PETase (Son et al., 2020). Furthermore, site-directed mutagenesis (SDM) has efficiently been applied in degradation studies. Usually, an *Escherichia coli* strain is used to carry plasmid-encoding mutant and to express desirable enzymes. Furukawa et al. (2019) showed that the hydrolysis activity of mutant thermostable cutinase from *T. fusca* (TfCut2) expressed in the *E. coli* model system was 12.7 times higher than the wild-type TfCut2. Hence, the application of SDM will find bioremediative potential against MPs.

Microalgae have been extensively studied for biotechnological applications, mainly to make biofuels. However, several studies have reported the bioremediative potential of microalgae as microbial chassis. In synthetic biology, a chassis is an organism that shelters and sustains genetic components by supplying resources needed for cellular functions (Chi et al., 2019). Numerous functional expression studies were conducted using a green alga (Kim et al., 2020) and a diatom (Moog et al., 2019). Therefore, using eukaryotic microalgae instead of bacteria as model systems provide a viable and eco-friendly method for the bioremediation of microplastic-polluted water.

## 4. Discussion

Plastic production has been increasing for the past decades due to the high demands of different sectors. Anthropogenic activities and improper waste disposal are the leading causes of rampant plastic pollution in the environment. Since the COVID-19 pandemic occurred in 2020, global plastic pollution has increased (Ammendolia et al., 2021), which could escalate the number of MPs (Liang et al., 2022). Thus, microplastic distribution is an emerging environmental issue that needs a long-term and sustainable solution. Bioremediation is a sustainable method to mitigate quantities of plastic contaminants, including MPs. Numerous studies stated the promising application of microbe-enzyme systems for the bioremediation of pollutants. Some of the prospective microbes are yeast (Tkavc et al., 2018), algae (Rehman et al., 2006), fungi (García-Delgado et al., 2015), and bacteria (Fu et al., 2021). This minireview paper concluded that the various enzymes of microbial origin have promising bioremediative applications in degrading synthetic microplastic particles.

Since enzymes could be produced extracellularly or intracellularly, studies focusing on intracellular enzymes are minimal. Nevertheless, a recent work published by Mohanan et al. (2022) demonstrated cloning and expression of intracellular lipases, which effectively hydrolyzed short- and medium-chain length plastics suggesting a potential bioremediative approach in microplastic biodegradation. Other innovative approaches, such as nanotechnology and enzyme immobilization, have started gaining attention for future applications to degrade MPs. The site-directed immobilization strategy for PETase on magnetic nanoparticles revealed a promising strategy for microplastic reduction (Schwaminger et al., 2021). Additionally, the application of microbial consortia in microplastic bioremediation is exceedingly encouraging. The synergistic actions of microbial consortia and enzymatic activities from various microbial networks should be thoroughly investigated. Enzyme cocktails also showed enhanced degrading action against complex polymers (Mekasha et al., 2016; Contreras et al., 2020), which can be considered an alternative option for microplastic bioremediation, especially for recalcitrant MPs. Given the seemingly endless potential of microorganisms and their constant adaptation to the changing environment, it is expected that further research in this area will soon lead to realistic biodegradation procedures that can be applied on a commercial scale.

## Author contributions

RD wrote the original draft, revised original draft, collected references, and constructed illustrations. RA edited the original draft, revised original draft, and supervised. All authors contributed to the article and approved the submitted version.

## Funding

This work was funded by the United Arab Emirates University Program for Advanced Research (2020-2022).

## Conflict of interest

The authors declare that the research was conducted in the absence of any commercial or financial relationships that could be construed as a potential conflict of interest.

## Publisher’s note

All claims expressed in this article are solely those of the authors and do not necessarily represent those of their affiliated organizations, or those of the publisher, the editors and the reviewers. Any product that may be evaluated in this article, or claim that may be made by its manufacturer, is not guaranteed or endorsed by the publisher.
